# Outlet type constipation in adult patients treated with type A botulinum toxin: a cohort study

**DOI:** 10.1007/s00384-024-04795-5

**Published:** 2025-01-21

**Authors:** Giuseppe Brisinda, Valeria Fico, Giuseppe Tropeano, Maria Cariati, Gaia Altieri, Filomena Misuriello, Gilda Pepe, Pietro Fransvea, Maria Michela Chiarello

**Affiliations:** 1 Catholic School of Medicine, “Agostino Gemelli”, 00168 Rome, Italy; 2 Department of Medical and Surgical Sciences, Fondazione Policlinico Universitario A Gemelli, IRCCS, 00168 Rome, Italy; 3 Department of Surgery, Azienda Sanitaria Provinciale Crotone, 88900 Crotone, Italy; 4 Department of Surgery, Azienda Sanitaria Provinciale Cosenza, 87100 Cosenza, Italy

**Keywords:** Botulinum toxin, Chronic constipation, Defecatory disorder, Outlet type constipation

## Abstract

**Purpose:**

Chronic constipation is a common symptom. Constipation due to pelvic floor disorders remain a therapeutic challenge. Biofeedback therapy is considered as the first-choice treatment for pelvic floor disorders, whenever dedicated expertise is available. Type A botulinum toxin has been used to selectively weaken the external anal sphincter and puborectalis muscle in constipated patients.

**Method:**

Eighty-two patients with chronic outlet obstruction constipation were treated with 100 units type A botulinum toxin, injected into the puborectalis muscle and the external anal sphincter.

**Results:**

At the 2-month evaluation, a symptomatic improvement was noted in 69 patients. Seven (8.5%) patients had mild flatus incontinence. Stool frequency per week increased from 2.4 ± 0.9 to 5.1 ± 1.0 (*P* = 0.0001). Anorectal manometry demonstrated decreased tone during straining from 91 ± 28 mmHg to 61 ± 27 mmHg (*P* = 0.0001). Defecography after the treatment showed improvement in anorectal angle during straining, which increased from 96 ± 12° to 124 ± 14° (*P* = 0.0001).

**Conclusion:**

Type A botulinum toxin relaxes the puborectalis muscle. Pressure values decline after the treatment. Transrectal ultrasonography to guide injections is a safe procedure. Repeated injections were needed to maintain the clinical improvement.

## Introduction

Chronic constipation is a common symptom [1, 2]. Recent published criteria for chronic constipation categorize patients as having functional form, constipation-predominant irritable bowel syndrome, or defecatory dysfunction [3–6].

The aetiology of defecatory disorders is characterized by a failure of the puborectalis muscle to relax during efforts to defecate or by its paradoxical contraction [6].

The treatment of this dysfunction is not easy to perform [7, 8]. Surgical treatment has often been unsuccessful [5]. Conceptually, biofeedback appears to be the most appropriate treatment, although several studies using biofeedback techniques have not documented consistent results [9, 10]. This is mainly related to which prevalent form of constipation this treatment is applied to. It is known that biofeedback therapy has little effect on patients with isolated slow transit constipation. On the other hand, about 70% of patients with defecation disorders benefit from biofeedback treatment. Despite this, a recent national evaluation concluded that there is insufficient evidence to conclusively confirm the efficacy of this therapy in patients with chronic idiopathic constipation [9].

Type A botulinum toxin has been used to selectively weaken the external anal sphincter and puborectalis muscle in constipated patients [11–14]. The toxin has also been used in constipated patients with associated neurological disorders such as Parkinson’s disease [15] or spinal cord injury [16]. It has been demonstrated that the toxin relaxes the puborectalis muscle [17]; consequently, the relaxation of the puborectalis muscle and the external anal sphincter allows the anorectal angle to increase during straining, facilitating evacuation [15, 18]. In this context, however, it must be emphasized that the optimal dose of toxin to be used in constipated adult patients is not well defined. Another aspect that should not be overlooked is the fact that different brands of type A botulinum toxin type show different biological activity [19]. Furthermore, the best method of toxin administration is not well defined. Palpation-guided injection enhanced resting anal pressure compared to endoanal ultrasound injection.

In our previous experiences, we have used type A botulinum toxin in adult constipated patients with promising results [11, 17]. We have used a dose of type A botulinum toxin ranging from 30 to 60 units. In this study, we present the clinical experience on the efficacy of 100 units of type A botulinum toxin injected under transrectal ultrasound guidance in improving rectal emptying in patients with defecatory disorders involving spastic pelvic floor muscles.

## Materials and methods

All patients who had symptoms of chronic constipation for the last 3 months, and no organic gastrointestinal pathology, were included in this cohort study performed at Fondazione Policlinico A Gemelli Istituto di Ricerca e Cura a Carattere Scientifico (IRCCS), in a 15-year period (2007–2021). This study follows the STROBE reporting guidelines. The patients have been categorized as functional defecation disorders when they showed abnormal anorectal evaluation pattern with anorectal manometry and impaired rectal evacuation on defecography. The inclusion and exclusion criteria for treatment with type A botulinum toxin are reported in Table 1.

**Table 1:** Inclusion and exclusion criteria

Inclusion criteria	1. Incomplete, prolonged and difficult evacuation with constant use of enemas, laxatives and manual maneuvers to facilitate bowel movement
	2. Age > 18 years
	3. Fewer than 3 bowel movement per week
	4. Straining during > 25% of the time
	5. Lumpy or hard stool > 25% of the time
	6. Sensation of anorectal obstruction > 25% of the time
	7. Sensation of incomplete evacuation > 25% of the time
	8. Manual maneuvers required to aid defecation > 25% of the time
	9. Normal colonic transit time study
	10. Failure to relax perineal floor during straining at physical examination
	11. Inability to achieve evacuation of barium paste during defecography, with the lack of a measurable increase in the anorectal angle between rest and attempted evacuation
	12. High pressure levels during straining at anorectal manometry;
	13. Absence of organic causes of constipation documented with colonoscopy and CT-scan
Exclusion criteria	1. Prevalent slow-transit constipation
	2. Constipation due to organic causes
	3. Previous surgical procedures on anal canal and rectum
	4. Previous treatment with type A botulinum toxin
	5. Concomitant oral medications that could interfere with the action of type A botulinum toxin, such as aminoglycosides, baclofen, dantrolene or diazepam
	6. Known hypersensitivity to any of the components of formulation of type A toxin
	7. Pregnant or breast-feeding women
	8. Rectocele at physical examination
	9. Rectocele larger than 3 cm in diameter as measured during defecography

### Study design

Each patient provided written informed consent for the study and underwent a baseline evaluation. All patients underwent anorectal manometry and defecography radiography, as described in previous experiences [11]. Two months after treatment, patients underwent the same assessment as performed at baseline.

### Treatment

Vials containing 100 units of type A botulinum toxin (Botox; Allergan, Inc, Irvine, CA, USA) were used. The vials were stored at − 20 °C and diluted in saline to 50 IU/ml before injections. Two ml were injected perineally with a 23-gauge needle. The two injection sites, on either side of the paradoxically contracting puborectalis muscle, were visualized by transrectal ultrasound. No sedation or local anaesthesia was used during toxin injection.

### Aim of the study

The study endpoint was the evaluation of symptom improvement after treatment with botulinum toxin. Secondary objectives are evaluations of the anorectal angle and anal canal pressure during straining.

### Follow-up

At the 2-month evaluation, if the symptoms persisted, the examiners could decide to re-treat a patient (rescue treatment) with 200 units of botulinum toxin, as described above. Then, the patients were evaluated with the same protocol 2 months after the rescue treatment. Control was carried out by telephone interviews (once a year). Clinical visit was performed only in patients who complained of specific symptoms.

### Statistical analysis

All statistical elaborations were obtained by using StatPlus for Mac (StatPlus, AnalystSoft, Inc., USA). The results were expressed as mean ± standard deviation (± SD) and compared by the Student’s *t*-test. All *P*-values were two-tailed. *P-*values of less than 0.05 were considered statistically significant.

## Results

During the study period, 82 patients were enrolled. Baseline characteristics are reported in Table 2. Type A botulinum toxin injections were performed easily in all patients. No complications or adverse events were observed during the procedure in any patient.

**Table 2:** Characteristics of 82 patients treated with type A botulinum toxin at baseline

Characteristics^a^		
Demographic and clinical data	M/F	37/45
	Age (years)	59.1 ± 13.4
	Duration of symptoms (months)	31.4 ± 13.7
	Stool frequency per week	2.4 ± 0.9
Anorectal manometry (mmHg)	Resting anal pressure	79 ± 15
	Voluntary contraction	62 ± 19
	Pressure during straining	91 ± 28
Defecography (degrees)	Anorectal angle at rest	115 ± 10
	Anorectal angle during straining	96 ± 12

^a^Values are given as mean ± SD. All patients were included in all evaluations

At the 2-month evaluation, inspection revealed a symptomatic improvement in 69 (84.1%) patients. Stool frequency per week increased from 2.4 ± 0.9 to 5.1 ± 1.0 (*P* = 0.0001). Seven (8.5%) patients had mild flatus incontinence. As compared with baseline values, pressure during straining was reduced by 32% (Table 3). Pressure during straining, at baseline higher than resting pressure (91 ± 28 mmHg vs 79 ± 15 mmHg, *P* = 0.001), decreased at the 2-month evaluation (61 ± 27 mmHg vs 75 ± 22 mmHg, *P* = 0.0004). As compared with baseline values, anorectal angle measured during straining increased by 27% (Table 4).

**Table 3:** Anal pressures before and after treatment with type A botulinum toxin in 82 patients treated with type A botulinum toxin

Parameters^a^	Baseline	2 months	*P* values^b^
Resting anal pressure	79 ± 15	75 ± 22	0.1
Voluntary contraction	62 ± 19	51 ± 21	0.0005
Pressure during straining	91 ± 28	61 ± 27	0.0001

^a^Values are given as mean ± SD. All patients were included in all evaluations. ^b^For the comparison with the baseline values (Student’s *t*-test)

**Table 4:** Results of defecography before and after treatment with type A botulinum toxin in 82 patients treated with type A botulinum toxin

Parameters^a^	Baseline	2 months	*P* values^b^
Anorectal angle at rest	115 ± 10	114 ± 9	0.5
Anorectal angle during straining	96 ± 12	124 ± 14	0.0001

^a^Values are given as mean ± SD. All patients were included in all evaluations. ^b^For the comparison with the baseline values (Student’s *t*-test)

At the 2-month evaluation, a salvage treatment was proposed to 13 symptomatic patients. This treatment consisted of the injection of 200 units of type A botulinum toxin with the same modalities previously described. Two months after the rescue treatment, symptomatic improvement was observed in all these patients. Stool frequency per week increased from 3.1 ± 0.6 to 4.9 ± 2.2 (*P* = 0.008). Of these patients 3 (23.1%) had flatus incontinence. At the same time, anorectal angle during straining was increased in these patients from 88 ± 13° to 126 ± 9° (30% increase, *P* = 0.0001), and the pressure during straining was reduced from 97 ± 29 mmHg to 61 ± 21 mmHg (37% decrease, *P* = 0.001). Pressure during straining decreased with respect to resting anal pressure (83 ± 19 mmHg, *P* = 0.01).

The patients were followed up for an average 30 months. During this time, 21 patients were lost to follow-up. Six patients showed relapse of symptoms 14 months after the treatment. Of these patients, two refused further treatment, while the other 4 were treated with 100 units of toxin. No complications or side effects were reported during follow up period from the remaining 55 patients.

## Discussion

Pelvic floor dyssynergia is a common cause of chronic constipation, hallmarked by inappropriate, paradoxical contraction, or a failed relaxation of the puborectalis muscle and external anal sphincter during defecation [14, 18, 20, 21]. Treatment of defecatory dysfunction is not always easy and rapidly effective [22, 23]. Conceptually, biofeedback training is the most logical approach to pelvic floor dyssynergia, although data from controlled studies are lacking [24, 25]. The success rate of biofeedback therapy for pelvic floor dysfunction varies widely between 40 and 90% [5, 26]. Randomized controlled trials have shown biofeedback to be superior to standard therapy (i.e., laxatives) in the treatment of this clinical condition [27, 28]. The limitations of biofeedback for dyssynergic defecation pertain to its availability in selected centres only and the need for multiple clinic visits [19]. A recent randomized controlled trial found home-based biofeedback to improve bowel symptoms and physiology similar to office-based biofeedback; this cost-effective approach may substantially broaden the availability and the use of this treatment [29].

Alternative therapies that produce a reversible reduction of anal canal pressure during straining, inducing an inhibition of the paradoxical puborectalis contraction, may be effective [14, 30]. Among these therapies, the injection of botulinum toxin, both in striated muscles and in smooth sphincters, has proven effective in the treatment of many diseases of the gastrointestinal tract. However, there are a limited number of studies evaluating the use of type A botulinum toxin in the treatment of pelvic floor dyssynergia in adult patients (Table 5) [13, 31, 32]. Among the many published guidelines on the diagnosis and treatment of chronic idiopathic constipation, only the French guidelines report the use of botulinum toxin for the treatment of this disease [26]. The same French guidelines summarize that the toxin induces a reduction in anal resting pressure and improves the relaxation of the puborectalis during straining, emphasizing that adverse events are negligible [33]. Recently, in a review of 189 patients, the median dose of type A botulinum toxin injected per procedure was 100 units, administered in most cases in the lateral area. A symptomatic improvement was documented in 77.4% at the 2-month evaluation and in 46% at 4 months after type A botulinum toxin injection. Complications were observed in a variable percentage, from 0 to 22.6%. The authors therefore emphasize that, although the symptomatic improvement is satisfactory, the clinical benefit worsens 3 months after the injection. The clinical benefit can be maintained with repeated injections, which appear to have no increase in morbidity [13].

**Table 5:** Published results of treatment of pelvic floor dyssynergia with type A botulinum toxin in adult patients

Author	Patients	Drug/dose	Symptomatic Improvement	Other results
Hallan et al.[35]	7	Dysport/Nr	57.1%	Maximum voluntary contraction from 70 to 28 cm H_2_O. Anorectal angle from 96 to 124°. Incontinence in 2 patients
Joo et al.[12]	4	Botox/6–15 U	100%	Two patients relapsed. No complications were reported
Shafik et al.[36]	15	Botox/25 U	86.7%	No complications were reported
Maria et al.[17]	4	Botox/30 U	75%	Anal tone during straining from 96.2 to 42.5 mmHg at 4 weeks and to 63.2 mmHg at 8 weeks. Anorectal angle from 94° to 114°. No complications were reported
Ron et al.[37]	25	Botox/20 U	75%	Perianal pain in 3 patients
Maria et al.[11]	24	Botox/60 U	79.2%	Anorectal manometry demonstrated decreased tone during straining from 98 ± 24 mmHg to 56 ± 20 mmHg at 1 month evaluation (*P* < 0.01) and 56 ± 29 mmHg at 2 months follow-up (*P* < 0.01). Defecography after the treatment showed improvement in anorectal angle during straining. No complications were reported
Farid et al.[31]	24	Dysport/100 U	70.8%	Improvement persisted only in 8 (33.3%). No complications were reported
Farid et al.[38]	15	Dysport/100 U	86.7%	Long-term success persisted only in six patients (40%). No complications were reported
Farid et al.[39]	20	Dysport/100 U	75%	Clinical improvement at 1 year 35%. Complications not reported
Hompes et al.[34]	56	Botox/100 U	96%	Isolated obstructed defecation symptoms, but not proctographic or physiological factors, predicted response on logistic regression analysis. In 33 (97%) of 34 non-responders, significant abnormalities were demonstrated: 31 (94%) had a grade 3–5 rectal prolapse, one had internal anal sphincter myopathy, and one had a fissure. Exclusion of these alternative diagnoses revised the initial response rate to 96%
Zhang et al.[32]	31	Xeomin/100 U	77.4%	After treatment, the pressure of the anal canal during rest and defecation was significantly reduced from 93 ± 16.5 mmHg and 105 ± 28.3 mmHg to 63 ± 8.6.3 mmHg and 42 ± 8.9 mmHg, respectively. Fecal incontinence in 8 patients

The current study provides further evidence of the usefulness of type A botulinum toxin in the management of pelvic floor dysfunction. In our experience, early symptomatic improvement was achieved after treatments, and there were no observable permanent complications. In the present study, we observed 7 cases of mild flatus incontinence after administration of 100 units of toxin. The incidence of mild flatus incontinence increased to 23.1% in the 13 patients treated with 200 units of type A botulinum toxin. High-dose of type A toxin significantly enhanced long-term improvement but also led to a significant higher rate of complications in comparison with low-dose toxin.

Compared with previous trials [11, 17], the better outcomes presented in this study could be a result of injection technique under ultrasonographic guidance. In the present series, also, we have been used higher doses of botulinum toxin than reported in our previous papers [11, 17]. Moreover, we selected patients more carefully and excluded from treatment patients with associated neurological pathologies or patients with anterior rectocele.

Careful patient selection is considered essential by Hompes et al. [34]. The authors treated 56 patients with 100 units of type A botulinum toxin. An initial favourable response was observed in 22 patients (39%). The authors also observed that solitary obstructed defecation symptoms predicted response on logistic regression analysis. In fact, excluding from the analysis non-responders who presented high-grade rectal prolapse, internal anal sphincter myopathy or even chronic anal fissure, the authors observed that the initial response was 96% [34]. With the same dose of toxin, we observed a symptomatic improvement in 84.1% of patients after a single administration.

The clinical response to botulinum toxin treatment is influenced by multiple factors. Among these, the dose used, and the method used for injection seem to have a significant importance. In this group of patients, we used a more precise method of injection that helped optimize the diffusion of the toxin. Diffusion of botulinum neurotoxin in tissues is also a dose-dependent phenomenon strongly linked to the dilution volume used. As compared with baseline values, pressure during straining was lower than the pre-treatment value and the resting anal pressure. The reduction of anal canal pressure during straining is directly related to the dose of injected toxin (32% reduction after 100 units of toxin and 37% reduction after 200 units). The increase in the anorectal angle during straining, also, related to the dose of injected toxin (27% after 100 units and 30% after 200 units of toxin), determined an increase in the stool frequency per week. In each case, in absence of any other treatment, these results could be attributed only to the effect of type A botulinum toxin. Furthermore, we have noted that, despite a more precise method of botulinum toxin injection, repeated injections were needed to maintain clinical improvement.

## Conclusions

In summary, despite the limitations of this study (single center, no comparison with other therapies), we can consider that type A botulinum toxin is an effective therapy for the treatment of adult patients with defecatory dysfunction. The toxin causes a resolution of the contraction of the puborectalis muscle.

The injection of toxin is easy to perform, and it is performed in an outpatient setting, without the need for hospitalization and/or sedation or anesthesia. The treatment is well tolerated by the patient, who undergoes this therapy more easily than biofeedback training. The injection of toxin does not depend on the patient’s willingness to complete the treatment.

Some aspects of this treatment still need to be improved, in particular the duration of the effectiveness of the injections, the standardization of repeated treatments, and the optimal dose of type A botulinum toxin to be administered.

## Data Availability

No datasets were generated or analysed during the current study.

## References

[1] Lacy BE, Xu Y, Taylor DCA, Kosch KJ, Dobrescu R, Morlock A et al (2024) Treatment for chronic idiopathic constipation: use and satisfaction from a nationwide survey of US participants. Neurogastroenterol Motil 36:e14885 39155456 10.1111/nmo.14885

[2] Ishibashi K, Urabe Y, Vu NTH, Miyauchi S, Nakamura T, Konishi H et al (2024) Clinical factors associated with stable treatment of chronic constipation in Japanese patients. BMC Gastroenterol 24:52 38287249 10.1186/s12876-024-03140-y PMC10823644

[3] Drossman DA (2016) Functional gastrointestinal disorders: history, pathophysiology, clinical features and Rome IV. Gastroenterology. 10.1053/j.gastro.2016.02.032 27144617 10.1053/j.gastro.2016.02.032

[4] Camilleri M, Ford AC, Mawe GM, Dinning PG, Rao SS, Chey WD et al (2017) Chronic constipation. Nat Rev Dis Primers 3:17095 29239347 10.1038/nrdp.2017.95

[5] Bharucha AE, Lacy BE (2020) Mechanisms, evaluation, and management of chronic constipation. Gastroenterology 158:1232–49 e3 31945360 10.1053/j.gastro.2019.12.034 PMC7573977

[6] Rao SSC, Brenner DM (2021) Efficacy and safety of over-the-counter therapies for chronic constipation: an updated systematic review. Am J Gastroenterol 116:1156–1181 33767108 10.14309/ajg.0000000000001222 PMC8191753

[7] Wald A, Bharucha AE, Cosman BC, Whitehead WE (2014) ACG clinical guideline: management of benign anorectal disorders. Am J Gastroenterol 109:1141–57 25022811 10.1038/ajg.2014.190

[8] Schmulson MJ, Drossman DA (2017) What Is New in Rome IV. J Neurogastroenterol Motil 23:151–163 28274109 10.5056/jnm16214 PMC5383110

[9] Etherson KJ, Horrocks EJ, Scott SM, Knowles CH, Yiannakou Y (2017) A national biofeedback practitioners service evaluation: focus on chronic idiopathic constipation. Frontline Gastroenterol 8:62–67 28839886 10.1136/flgastro-2015-100660 PMC5369454

[10] Woodward S, Norton C, Chiarelli P (2014) Biofeedback for treatment of chronic idiopathic constipation in adults. Cochrane Database Syst Rev (3):CD008486. 10.1002/14651858.CD008486.pub2 10.1002/14651858.CD008486.pub2 PMC10618629 24668156

[11] Maria G, Cadeddu F, Brandara F, Marniga G, Brisinda G (2006) Experience with type A botulinum toxin for treatment of outlet-type constipation. Am J Gastroenterol 101:2570–2575 17029615 10.1111/j.1572-0241.2006.00791.x

[12] Joo JS, Agachan F, Wolff B, Nogueras JJ, Wexner SD (1996) Initial North American experience with botulinum toxin type A for treatment of anismus. Dis Colon Rectum 39:1107–1111 8831524 10.1007/BF02081409

[13] Emile SH, Elfeki HA, Elbanna HG, Youssef M, Thabet W, Abd El-Hamed TM et al (2016) Efficacy and safety of botulinum toxin in treatment of anismus: a systematic review. World J Gastrointest Pharmacol Ther 7:453–462 27602248 10.4292/wjgpt.v7.i3.453 PMC4986396

[14] Cariati M, Chiarello MM, Cannistra M, Lerose MA, Brisinda G (2021) Gastrointestinal uses of botulinum toxin. Handb Exp Pharmacol 263:185–226 32072269 10.1007/164_2019_326

[15] Cadeddu F, Bentivoglio AR, Brandara F, Marniga G, Brisinda G, Maria G (2005) Outlet type constipation in Parkinson’s disease: results of botulinum toxin treatment. Aliment Pharmacol Ther 22:997–1003 16268975 10.1111/j.1365-2036.2005.02669.x

[16] Valles M, Albu S, Kumru H, Mearin F (2021) Botulinum toxin type-A infiltration of the external anal sphincter to treat outlet constipation in motor incomplete spinal cord injury: pilot cohort study. Scand J Gastroenterol 56:777–783 34000949 10.1080/00365521.2021.1921255

[17] Maria G, Brisinda G, Bentivoglio AR, Cassetta E, Albanese A (2000) Botulinum toxin in the treatment of outlet obstruction constipation caused by puborectalis syndrome. Dis Colon Rectum 43:376–380 10733120 10.1007/BF02258305

[18] Maria G, Sganga G, Civello IM, Brisinda G (2002) Botulinum neurotoxin and other treatments for fissure-in-ano and pelvic floor disorders. Br J Surg 89:950–961 12153619 10.1046/j.1365-2168.2002.02121.x

[19] Chu CY, Su YC, Hsieh PC, Lin YC (2023) Effectiveness and safety of botulinum neurotoxin for treating dyssynergic defecation: a systematic review and meta-analysis. Toxicon 235:107311 37816487 10.1016/j.toxicon.2023.107311

[20] Bove A, Pucciani F, Bellini M, Battaglia E, Bocchini R, Altomare DF et al (2012) Consensus statement AIGO/SICCR: diagnosis and treatment of chronic constipation and obstructed defecation (part I: diagnosis). World J Gastroenterol 18:1555–1564 22529683 10.3748/wjg.v18.i14.1555 PMC3325520

[21] Schiller LR (2019) Chronic constipation: new insights, better outcomes? Lancet Gastroenterol Hepatol 4:873–882 31609241 10.1016/S2468-1253(19)30199-2

[22] Camilleri M (2011) New treatment options for chronic constipation: mechanisms, efficacy and safety. Can J Gastroenterol 25(Suppl B):29–35 PMC3206563 22114755

[23] Shin JE, Jung HK, Lee TH, Jo Y, Lee H, Song KH et al (2016) Guidelines for the diagnosis and treatment of chronic functional constipation in Korea, 2015 Revised Edition. J Neurogastroenterol Motil 22:383–411. 10.5056/jnm15185 PMC4930295 27226437

[24] Chiarioni G (2016) Biofeedback treatment of chronic constipation: myths and misconceptions. Tech Coloproctol 20:611–618 27450533 10.1007/s10151-016-1507-6

[25] Quigley EM, Neshatian L (2016) Advancing treatment options for chronic idiopathic constipation. Expert Opin Pharmacother 17:501–511 26630260 10.1517/14656566.2016.1127356

[26] Vitton V, Damon H, Benezech A, Bouchard D, Brardjanian S, Brochard C et al (2018) Clinical practice guidelines from the French National Society of Coloproctology in treating chronic constipation. Eur J Gastroenterol Hepatol 30:357–363 29406436 10.1097/MEG.0000000000001080

[27] Chiarioni G, Whitehead WE, Pezza V, Morelli A, Bassotti G (2006) Biofeedback is superior to laxatives for normal transit constipation due to pelvic floor dyssynergia. Gastroenterology 130:657–664 16530506 10.1053/j.gastro.2005.11.014

[28] Corsetti M, Brown S, Chiarioni G, Dimidi E, Dudding T, Emmanuel A et al (2021) Chronic constipation in adults: contemporary perspectives and clinical challenges. 2: Conservative, behavioural, medical and surgical treatment. Neurogastroenterol Motil 33:e14070 33522079 10.1111/nmo.14070

[29] Rao SSC, Valestin JA, Xiang X, Hamdy S, Bradley CS, Zimmerman MB (2018) Home-based versus office-based biofeedback therapy for constipation with dyssynergic defecation: a randomised controlled trial. Lancet Gastroenterol Hepatol 3:768–777 30236904 10.1016/S2468-1253(18)30266-8 PMC6206847

[30] Chaichanavichkij P, Vollebregt PF, Scott SM, Knowles CH (2020) Botulinum toxin type A for the treatment of dyssynergic defaecation in adults: a systematic review. Colorectal Dis 22:1832–1841 32403161 10.1111/codi.15120

[31] Farid M, El Monem HA, Omar W, El Nakeeb A, Fikry A, Youssef T et al (2009) Comparative study between biofeedback retraining and botulinum neurotoxin in the treatment of anismus patients. Int J Colorectal Dis 24:115–120 18719924 10.1007/s00384-008-0567-0

[32] Zhang Y, Wang ZN, He L, Gao G, Zhai Q, Yin ZT et al (2014) Botulinum toxin type-A injection to treat patients with intractable anismus unresponsive to simple biofeedback training. World J Gastroenterol 20:12602–12607 25253964 10.3748/wjg.v20.i35.12602 PMC4168097

[33] Bassotti G, Usai Satta P, Bellini M (2021) Chronic idiopathic constipation in adults: a review on current guidelines and emerging treatment options. Clin Exp Gastroenterol 14:413–428 34712055 10.2147/CEG.S256364 PMC8547593

[34] Hompes R, Harmston C, Wijffels N, Jones OM, Cunningham C, Lindsey I (2012) Excellent response rate of anismus to botulinum toxin if rectal prolapse misdiagnosed as anismus ('pseudoanismus’) is excluded. Colorectal Dis 14:224–230 21689279 10.1111/j.1463-1318.2011.02561.x

[35] Hallan RI, Williams NS, Melling J, Waldron DJ, Womack NR, Morrison JF (1988) Treatment of anismus in intractable constipation with botulinum. A toxin Lancet 2(8613):714–717 2901570 10.1016/s0140-6736(88)90188-2

[36] Shafik A, El-Sibai O (1998) Botulin toxin in the treatment of nonrelaxing puborectalis syndrome. Dig Surg 15(4):347–351. 10.1159/000018630 9845612 10.1159/000018630

[37] Ron Y, Avni Y, Lukovetski A, Wardi J, Geva D, Birkenfeld S, Halpern Z (2001) Botulinum toxin type-A in therapy of patients with anismus. Dis Colon Rectum 44(12):1821–1826. 10.1007/BF02234461 11742168 10.1007/BF02234461

[38] Farid M, Youssef T, Mahdy T, Omar W, Moneim HA, El Nakeeb A, Youssef M (2009) Comparative study between botulinum toxin injection and partial division of puborectalis for treating anismus. Int J Colorectal Dis 24(3):327–334. 10.1007/s00384-008-0609-7 19039596 10.1007/s00384-008-0609-7

[39] Farid M, El Nakeeb A, Youssef M, Omar W, Fouda E, Youssef T, Thabet W, Elmoneum HA, Khafagy W (2009) Idiopathic hypertensive anal canal: a place of internal sphincterotomy. J Gastrointest Surg 13(9):1607–1613. 10.1007/s11605-009-0931-6 19517198 10.1007/s11605-009-0931-6

